# Urgent transcatheter edge-to-edge repair for severe mitral regurgitation with flail leaflet in critically Ill patients

**DOI:** 10.3389/fcvm.2023.1197345

**Published:** 2023-06-16

**Authors:** Nimrod Perel, Itshak Amsalem, Or Gilad, Rafael Hitter, Tomer Maller, Elad Asher, Emanuel Harari, David Marmor, Shemy Carasso, Danny Dvir, Michael Glikson, Mony Shuvy

**Affiliations:** ^1^ Jesselson Integrated Heart Center, Shaare Zedek Medical Center and Faculty of Medicine, Hebrew University, Jerusalem, Israel; ^2^ The Azrieli Faculty of Medicine, Bar Ilan University, Safed, Israel

**Keywords:** acute TEER, flail leaflet, ruptured chordae, acute mitral regurgitation, criticall illness

## Abstract

**Introduction:**

Degenerative mitral valve disease (DMR) is a common valvular disorder, with flail leaflets due to ruptured chordae representing an extreme variation of this pathology. Ruptured chordae can present as acute heart failure which requires urgent intervention. While mitral valve surgery is the preferred mode of intervention, many patients have significantly elevated surgical risk and are sometimes considered inoperable. We aim to characterize patients with ruptured chordae undergoing urgent transcatheter edge-to-edge repair (TEER), and to analyze their clinical and echocardiographic outcomes.

**Methods:**

We screened all patients who underwent TEER at a tertiary referral center in Israel. We included patients with DMR with flail leaflet due to ruptured chordae and categorized them into elective and critically ill groups. We evaluated the echocardiographic, hemodynamic, and clinical outcomes of these patients.

**Results:**

The cohort included 49 patients with DMR due to ruptured chordae and flail leaflet, who underwent TEER. Seventeen patients (35%) underwent urgent intervention and 32 patients (65%) underwent an elective procedure. In the urgent group, the average age of the patient was 80.3, with 41.8% being female. Fourteen patients (82%) received noninvasive ventilation, and three patients (18%) required invasive mechanical ventilation. One patient died due to tamponade, while echo evaluation of the other 16 patients demonstrated successful reduction of ≥2 in the MR grade. Left atrial V wave decreased from 41.6 mmHg to 17.9 mmHg (*p* < 0.001), and the pulmonic vein flow pattern changed from reversal (68.8%) to a systolic dominant flow in all patients (*p* = 0.001). After the procedure, 78.5% of patients improved to New York Heart Association (NYHA) class I or II (*p* < 0.001). There was no significant difference in the overall mortality between the urgent and elective groups, with similar 6 months survival rates for each group.

**Conclusion:**

Urgent TEER in patients with ruptured chordae and flail leaflets can be safe and feasible with favorable hemodynamic, echocardiographic, and clinical outcomes.

## Introduction

Primary mitral regurgitation is a common valvular condition in which a primary lesion in any component of the valve apparatus can lead to mitral regurgitation (MR) (1).

MR is the second most common valvular disorder, with primary MR being almost twice as common as secondary MR. Degenerative mitral regurgitation (DMR) due to myxomatous degeneration has two distinct etiologies: fibroelastic deficiency with a focal lesion, or Barlow disease with diffuse involvement of both leaflets. The average age of patients with DMR is over 65. Patients with fibroelastic deficiency tend to be older with a more acute presentation, while patients with Barlow disease are younger and have a more chronic disease course (2, 3).

According to the Carpentier classification, DMR is categorized as Carpentier type 2 with leaflet or chordal degeneration leading to an excessive leaflet motion. Flail leaflet represents an extreme variation of this pathology, with excessive leaflet motion most frequently being caused by ruptured chordae. According to the American Society of Echocardiography (ASE) guidelines on non-invasive evaluation of native valvular regurgitation, flail leaflet is one of the specific criteria for severe MR and its presence often denotes severe MR (4, 5).

Patients with flail leaflets can either present acutely with pulmonary edema and cardiogenic shock, or chronically with signs and symptoms of chronic heart failure. Despite surgery being the preferred mode of intervention, many of these patients are at elevated surgical risk and are often deemed to be inoperable. When these patients undergo urgent surgical intervention, morbidity and mortality are significantly higher than in elective cases (6). As a result, many of these patients receive medical therapy alone. Transcatheter edge-to-edge repair (TEER) has been proven to be a safe and effective treatment option for patients with chronic primary MR (7). While data on the use of urgent TEER for acutely ill patients in cardiogenic shock with significant MR is scarce and originates from smaller cohorts, the results are encouraging and show improved survival rates and outcomes. However, most of the data on acute TEER intervention is based on functional MR, especially in the setting of acute myocardial infarction (8, 9). Data on the use of acute TEER for patients with degenerative MR due to acute ruptured chordae and flail leaflets are limited. We aim to focus on patients who presented with acute symptoms and underwent urgent TEER procedures. In addition, in order to better understand this patient group, we compared their echocardiographic and clinical outcomes to individuals undergoing elective procedures.

## Methods

### Study population

We screened all patients who underwent TEER from January 2020 until October 2022 and followed them until January 2023. All patients underwent the procedure at a single medical center that serves as a referral center for several hospitals across Israel and averages over 100 TEER procedures annually. Procedures are performed for both functional and degenerative MR, including patients who require urgent interventions.

Patients typically receive elective TEER procedures unless they develop HF symptoms that persist and necessitate urgent TEER intervention. In the current study, we focused on patients with severe MR caused by flail leaflets due to ruptured chordae, and categorized them into elective and critically ill groups. Patients in the elective group presented with signs and symptoms of chronic heart failure, and without acute decompensation necessitating urgent intervention. Patients in the urgent group were hospitalized for acute heart failure and underwent urgent TEER procedures during that hospitalization. Additional patient stratification was done based on severity, ranging from non-invasive ventilatory assistance to cardiogenic shock with mechanical circulatory support. All patients were at high surgical risk and were approved for TEER after discussion by the institution's multidisciplinary heart team.

### Echocardiographic assessment

Transthoracic echocardiography (TTE) was initially performed in all patients to assess the severity of the MR. Subsequently, all patients underwent transesophageal echocardiography (TEE) to further evaluate the mechanism of the regurgitation and to assess their suitability for TEER. Evaluation of severe MR was based on current ASE guidelines and was graded from 1 to 4 as mild, moderate, moderate-severe, or severe. TTE was repeated the day after the procedure to reassess MR severity and measure post-procedural mean gradient.

### Intervention

Transcatheter edge-to-edge repair was performed by the implantation of a “MitraClip®” device (Abbott, Abbott Park, IL, USA) under fluoroscopic and TEE guidance. TEE was used for grading the severity of the MR and for decision-making during the procedure (the implantation of an additional clip or the ending of the procedure). At least one clip was implanted in all patients. Additional clips were implanted to achieve better MR reduction when deemed necessary by the operators. Left atrial V wave was measured and recorded before and after TEER implantation. Measurement after implantation was done while the TEE probe was still in place and before the transeptal delivery system was removed to provide reliable measurements of LA pressure. We calculated the absolute reduction in the Left atrial V wave before and after the procedure. Pulsed wave doppler assessment of pulmonic systolic vein flow was checked before and after clip implantation.

### Outcomes

We evaluated clinical outcomes including pre and post-procedure New York Heart Association (NYHA) functional class, and 30 and 60-days mortality.

Echocardiographic outcomes included: post-procedure mitral gradient, pulmonic vein flow systolic pattern (the appearance of a systolic dominance pattern after the procedure), grade of MR reduction, and tricuspid insufficiency jet velocities to estimate pulmonary artery systolic pressures.

### Statistical analysis

We evaluated all patients for their baseline characteristics and their echocardiographic and hemodynamic parameters. We compared the parameters between the urgent and elective groups before and after the procedure. We did additional assessment of the above parameters on the urgent TEER group.

Logical checks were performed on the data for quality control purposes. Normally distributed continuous variables are presented as means (±standard deviations) and non-normally distributed continuous variables are presented as medians (interquartile ranges).

Comparisons of independent continuous variables when assessing different subgroups (e.g., elective vs. emergent procedure), were done by using independent samples *T*-tests.

Comparisons of continuous variables before and after the procedures were carried out by using paired samples T-tests. Categorical variables were compared using the Chi-Square test. When necessary due to non-normal distributions, non-parametric tests were used.

All analyses were conducted using SPSS statistical software (version 26.0) and Rstudio (version 2022.07.2 + 576). All statistical tests were two-sided, and significance was determined at a *p*-value of 0.05.

## Results

**Figure 1:**
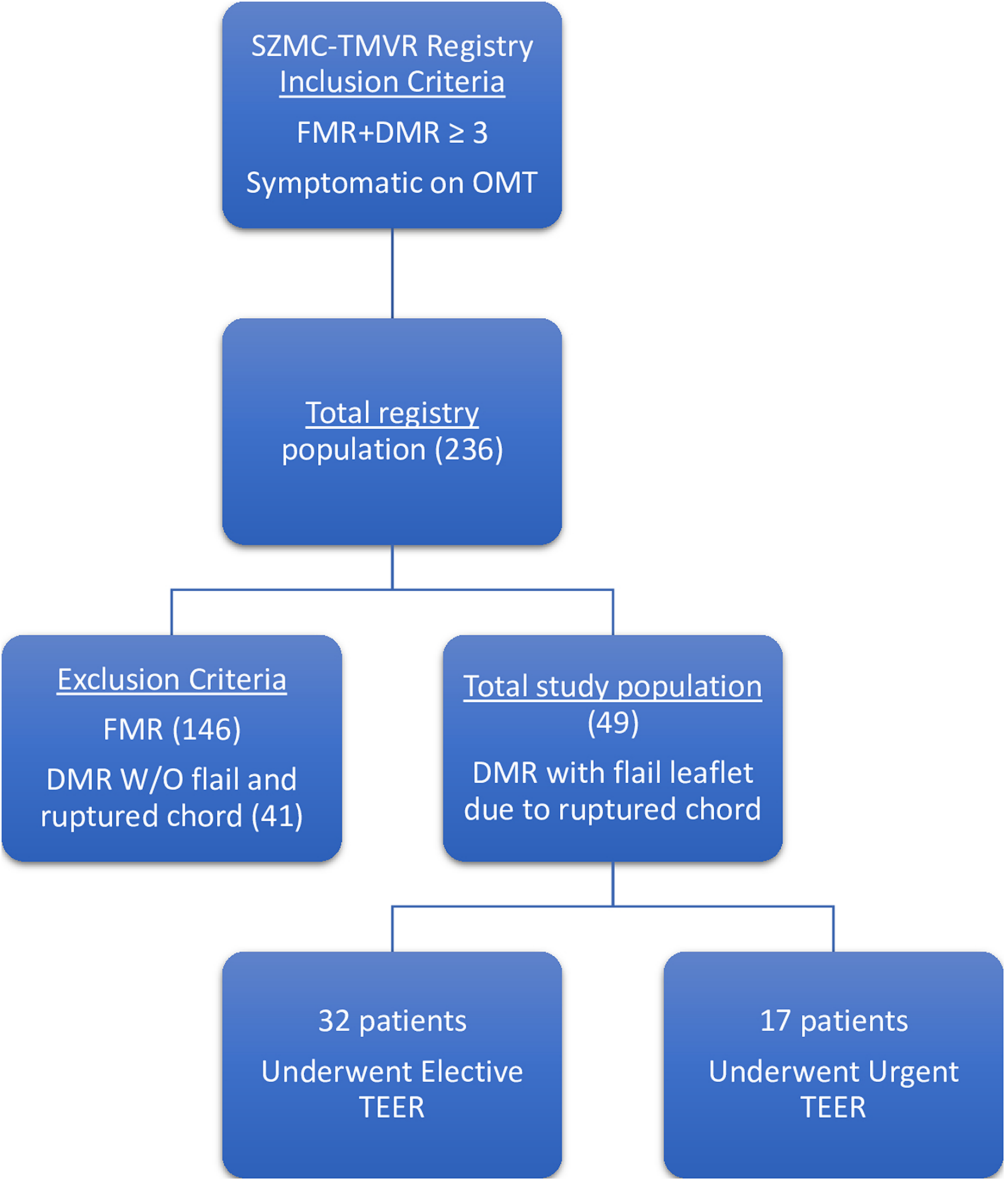
Trial inclusion criteria. SZMC -TMVR—Shaare Zedek medical center transcatheter mitral valve repair; FMR—functional mitral regurgitation; DMR—degenerative mitral regurgitation; OMT—optimal medical therapy; TEER—transcatheter edge to edge repair.

From a cohort of 236 patients who underwent TEER implantation between January 2020 and October 2022, we identified 49 patients who had DMR with ruptured chordae and flail leaflet. Patients with endocarditis or papillary muscle ruptured were excluded from the trial. Thirty-two patients out of 49 (65%) underwent an elective procedure, and 17 patients (35%) had TEER performed urgently during their hospitalization due to heart failure (Figure 1). Follow up time ranged between 3 and 36 months with a mean of 17.16 (±8.95) months.

The average age of the cohort was 79.7 ± 9 years, with 17 patients being female (34.6%). Hypertension was the most common comorbidity, (40 patients, 83.3%) followed by dyslipidemia and diabetes mellitus which were present in 29 and 11 patients respectively (60.4% and 22.9%). There was no significant difference seen in most of the baseline characteristics between the patients in the elective and urgent groups. There was a significant difference in the NYHA class between the two groups; all 17 patients in the urgent group were NYHA IV, compared with three patients (9.6% *p* < 0. 001) in the elective group (Table 1).

**Table 1:** Baseline characteristics.

		All (*N* = 49)	Elective (*N* = 32)	Urgent (*N* = 17)	*P* value
**Demographic**					
Age (yr) (mean [SD])		79.77 ± 9.02	79.49 ± 9.18	80.30 ± 8.96	0.769
Sex	Male—no. (%)	32 (65.31)	22 (68.75)	10 (58.82)	0.539
	Female—no. (%)	17 (34.69)	10 (31.25)	7 (41.18)	
**Medical history**					
Hypertension—no. (%)		40 (83.33)	26 (83.87)	14 (82.35)	1.00
Dyslipidemia—no. (%)		29 (60.42)	19 (61.29)	10 (58.82)	1.00
BMI (kg/m^2^) (mean [SD])		26.36 ± 6.37	25.20 ± 4.95	28.87 ± 8.41	0.099
IHD—no. (%)		20 (41.67)	14 (45.16)	6 (35.29)	0.555
Previous MI—no. (%)		11 (22.92)	9 (29.03)	2 (11.76)	0.284
Previous PCI—no. (%)		15 (31.25)	10 (32.26)	5 (29.41)	1.00
Previous CABG—no. (%)		3 (6.25)	3 (9.68)	0 (0.00)	–
NYHA	4—no. (%)	20 (40.8)	3 (9.68)	17 (100)	<0. 001
	Non-4—no. (%)	29 (60.42)	28 (90.32)	1 (5.88)	
AF—no. (%)		21 (43.75)	13 (41.94)	8 (47.06)	0.768
Previous Implantation of Pacemaker—no. (%)		3 (6.25)	2 (6.45)	1 (5.88)	1.00
DM—no. (%)		11 (22.92)	5 (16.13)	6 (35.29)	0.162
COPD—no (%)		14 (29.17)	12 (38.71)	2 (11.76)	0.094
OSA—no. (%)		15 (31.25)	12 (38.71)	3 (17.65)	0.196
Previous CVA or TIA—no. (%)		5 (10.42)	1 (3.23)	4 (23.53)	0.046
History of Smoking—no. (%)		6 (12.5)	2 (6.45)	4 (23.53)	0.166
History of Malignancy—no. (%)		13 (33.33)	8 (32.00)	5 (35.71)	1.00

BMI, body mass index; IHD, ischemic heart disease; MI, myocardial infarction; PCI, percutaneous coronary intervention; CABG, coronary artery bypass graft; NYHA, New York Heart Association; AF, atrial fibrillation; DM, diabetes mellitus; COPD, chronic obstructive pulmonary disease; OSA, obstructive sleep apnea; CVA, cerebrovascular accident; TIA, transient ischemic attack.

All patients in the urgent group needed ventilatory assistance. Thirteen patients (76.4%) were treated by non-invasive ventilatory support, while four patients (23%) received invasive mechanical ventilation. Two patients (11%) were in severe cardiogenic shock and required pharmacological and mechanical assistance. Cardiogenic shock was defined as systolic blood pressure <90 mmHg with signs of end organ hypoperfusion (10). The baseline echocardiographic parameters of the group included the presence of a flail in the posterior leaflet in 12 patients (70.5%), flail in the anterior leaflet in 5 patients (29.5%) and a bi-leaflet flail in one patient. Thirteen patients (76.4%) had A2-P2 pathology, and four patients (24.6%) had non-A2-P2 pathology. The average flail gap was 7.1 mm, with a range between 0.3 mm and 1.1 mm. Five patients (29.5%) had a Barlow morphology of their valve, with multi-segment billowing and multiple jets, as opposed to the other 12 patients (70.5%%) who had a non- Barlow morphology with a single segment involvement. 6 patients (35%) had multiple jets (all patients with Barlow morphology and additionally one more patient with a non-Barlow morphology). Significant valvular or subvalvular calcification was noted in 3 patients (17%).

**Figure 2:**
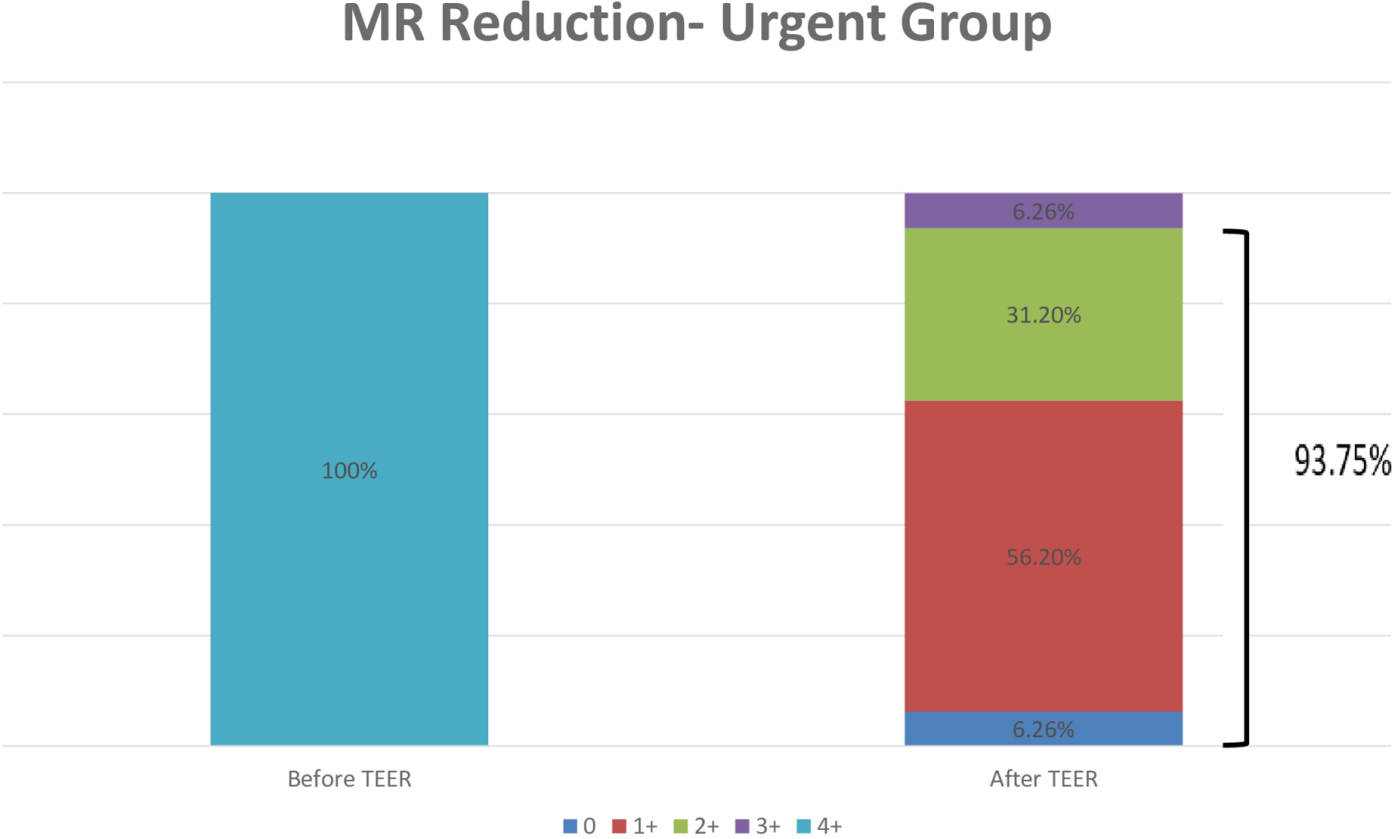
Transthoracic echocardiography evaluation of the MR grade before the procedure and 1 day after the procedure in the urgent group. MR- Mitral Regurgitation; TEER—transcatheter edge to edge repair.

**Figure 3:**
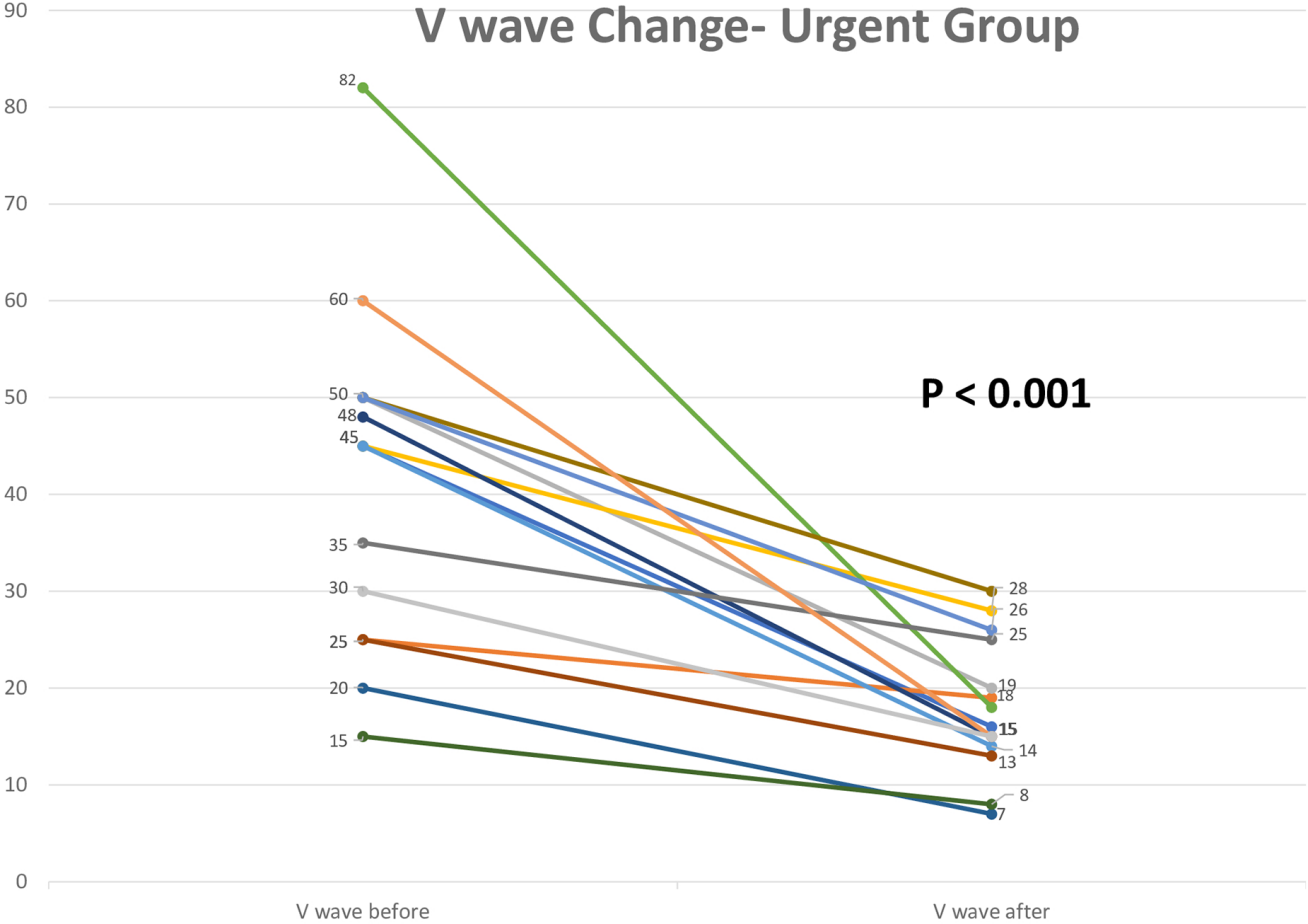
Left atrial V wave change before the procedure and immediately after clip implantation in the urgent group.

**Figure 4:**
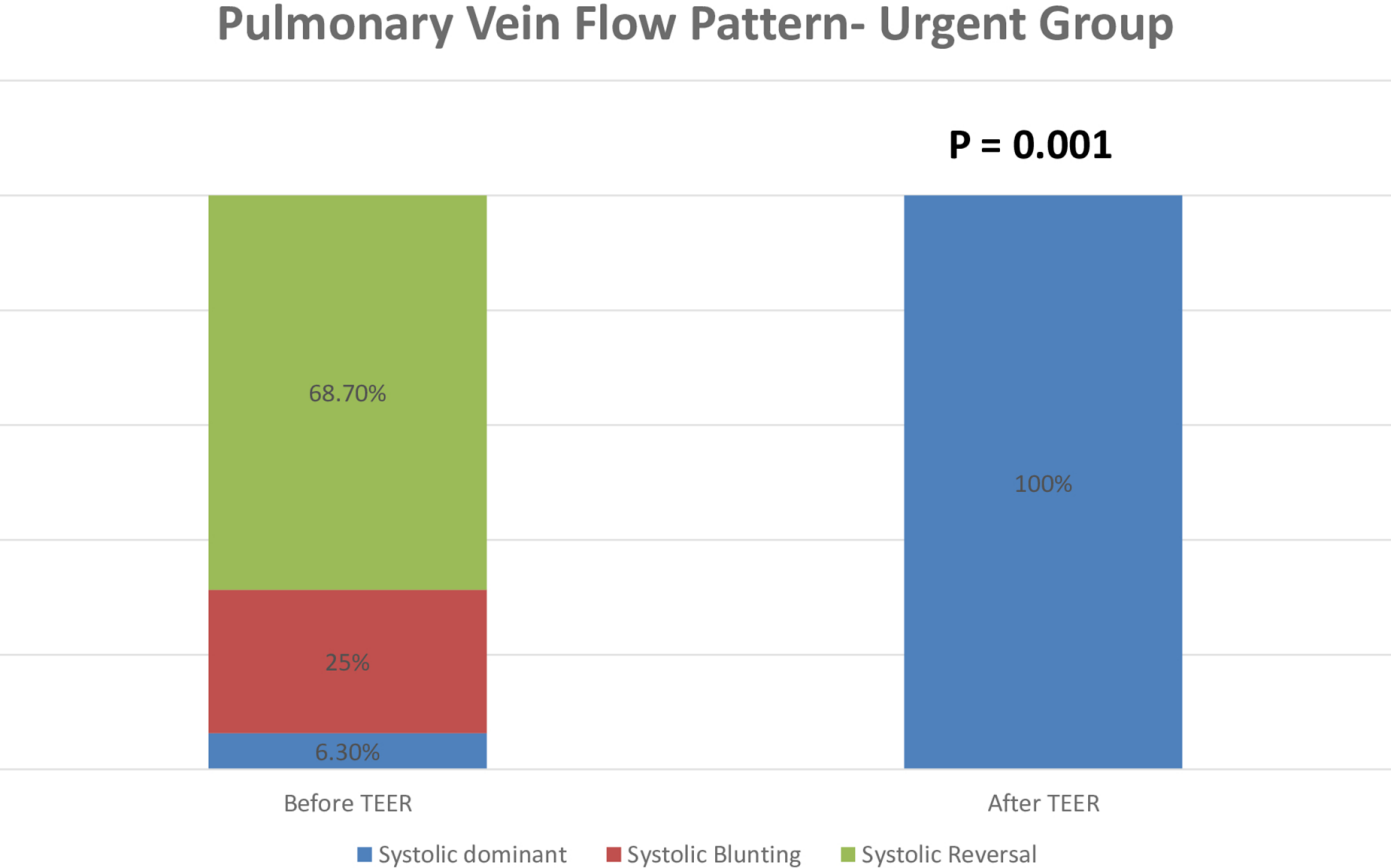
Transesophageal echocardiography evaluation of pulmonic vein flow pattern before the procedure and after the clip implantation in the urgent group. TEER—transcatheter edge to edge repair.

**Figure 5:**
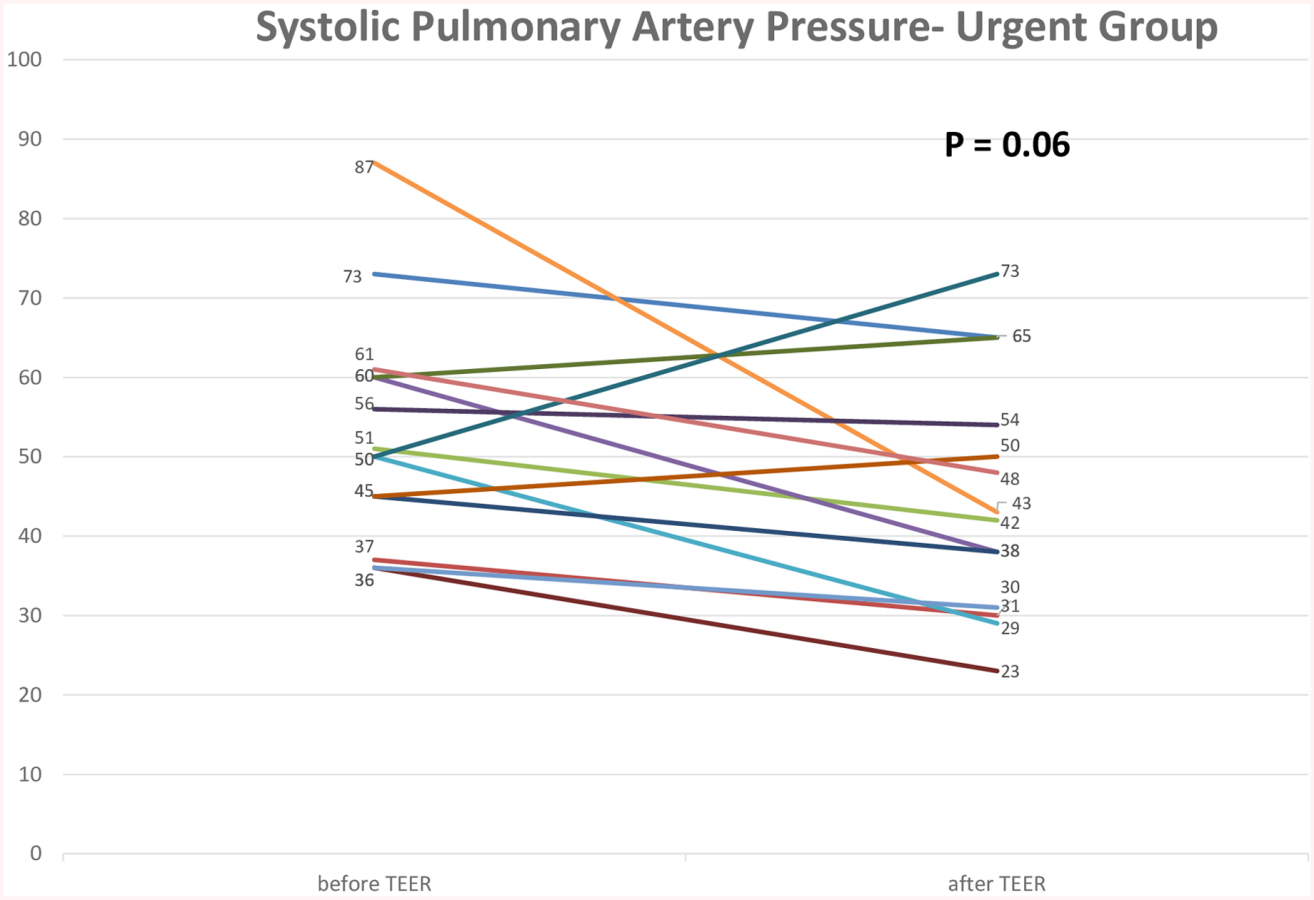
Transthoracic echocardiography evaluation of systolic pulmonary artery pressure before the procedure and at 1 day after the procedure in the urgent group. TEER—transcatheter edge to edge repair.

Postprocedural hemodynamic and echocardiographic parameters showed significant improvement in the MR grade across the entire cohort. All patients in the urgent group had a grade of +4 MR before the procedure, with a reduction to an average grade of +1.3 MR (Figure 2). In the paired analysis, the average left atrial V wave before the procedure was 41.6 mmHg, with a reduction to an average of 17.9 mmHg after the procedure (*p* < 0.001) (Figure 3). The pulmonic vein flow showed systolic flow reversal in 11 patients (68.8%) and systolic blunting in four patients (25%). The pulmonic vein flow doppler improved to systolic dominance in all patients after the procedure (*p* = 0.001) (Figure 4). The systolic pulmonary arterial pressure average was 52.6 mmHg before the procedure and decreased to 44.25 mmHg post-procedure (*p* = 0.06) (Figure 5). The improvement of the hemodynamic and echocardiographic parameters were similar in the elective group (Tables 2, 3). The average number of clips implanted was similar in both groups (1.69 in the elective group vs. 1.88 in the urgent group *p* = 0.28). In the urgent group, the type of clips used was 9 XTW (30.0%), 8 XT (26.6%), 7 NTW (23.4%), 6 NT (20.0%). In the urgent group, 12/16 patients (one patients died before implantation of the device) had 2 or more clips implanted. In twelve patients 2 or more clips were implanted. These patients were more likely to have a Barlow morphology of their valve, multiple jets or large coaptation gap usually required at least 2 clips implantation.

**Table 2:** Echocardiographic and hemodynamic parameters before TEER in All groups.

	All patients	Elective patients	Urgent patients	*P* Value
EF (%)	49	32	17	1.00
≥50%	44 (89.37%)	29 (90.33%)	15 (88.0%)	
31%—49%	5 (10.63%)	3 (9.67%)	2 (12.0%)	
≥30%	0 (0%)	0 (0%)	0 (0%)	
MR grade (%)				
4—Severe	49 (100%)	32 (100%)	17 (100%)	
sPAP mmHg (mean [SD])	51.53 (±16.57)	50.83 (±18.22)	52.60 (±14.22)	0.752
PV Flow (%)	43	27	16	0.016
1-Systolic Reversal	36 (83.7%)	25 (92.6%)	11 (68.8%)	
2- Systolic Blunting	6 (13.9%)	2 (7.4%)	4 (25%)	
3- Systolic Dominance	1 (2.3%)	0 (0%)	1 (6.2%)	
V wave mmHg (mean [SD])	40.47 (± 15)	39.90 (±14.32)	41.56 (±16.66)	0.724
Mitral valve gradient mmHg (mean [SD])	2.43 (± 1.47)	2.14 (±1.10)	3.0 (± 2.0)	0.216

EF, ejection fraction; MR, mitral regurgitation; sPAP, systolic pulmonary artery pressure; PV, pulmonic vein; SD, standard deviation.

**Table 3:** Echocardiographic and hemodynamic parameters after TEER in All groups.

	All patients	Elective patients	Urgent patients	*P* Value
MR grade (%)	49	32	17	0.498
0 -Trace	2 (4.3%)	1 (3.1%)	1 (6.2%)	
1- Mild	20 (40.4%)	10 (31.2%)	9 (56.2%)	
2—Moderate	22 (44.7%)	17 (53.1%)	5 (31.2%)	
3—Moderate Severe	4 (8.5%)	3 (9.3%)	1 (6.2%)	
4—Severe	1 (2.1%)	1 (3.1%)	0 (0%)	
sPAP mmHg (mean [SD])	43.13 (±14.43)	42.53 (±14.62)	44.25 (±14.46)	0.705
Delta sPAP mmHg (mean [SD])	−7.44 (± 15.97)	−6.82 (± 16.69)	−8.43 (± 15.33)	0.773
PV flow (%)	44	28	16	0.2
1—Systolic Reversal	1 (2.3%)	1 (3.6%)	0 (0%)	
2—Systolic Blunting	4 (9.1%)	4 (14.3%)	0 (0%)	
3—Systolic Dominance	39 (88.6%)	23 (82.1%)	16 (100%)	
Left atrial V wave mmHg (mean [SD])	19.9 (±7.08)	17.73 (±7.31)	17.93 (±6.84)	0.927
Delta Left atrial V wave mmHg (median [IQR])	−20 [17.0]	−20 [16.0]	−20 [19.0]	0.907
Mitral Valve Gradient mmHg (mean [SD])	5.14 (±2.73)	5.29 (±3.27)	4.86 (±1.21)	0.743
Delta Mitral Valve Gradient mmHg (mean [SD])	2.71 (±3.00)	3.14 (±3.48)	1.86 (±1.57)	0.368
Number of Clips Implanted Number of clips (mean (SD))	1.75 (0.56)	1.69 (0.53)	1.88 (0.62)	0.283

EF, ejection fraction; MR, mitral regurgitation; sPAP, systolic pulmonary artery pressure; PV, pulmonic vein; SD, standard deviation.

**Figure 6:**
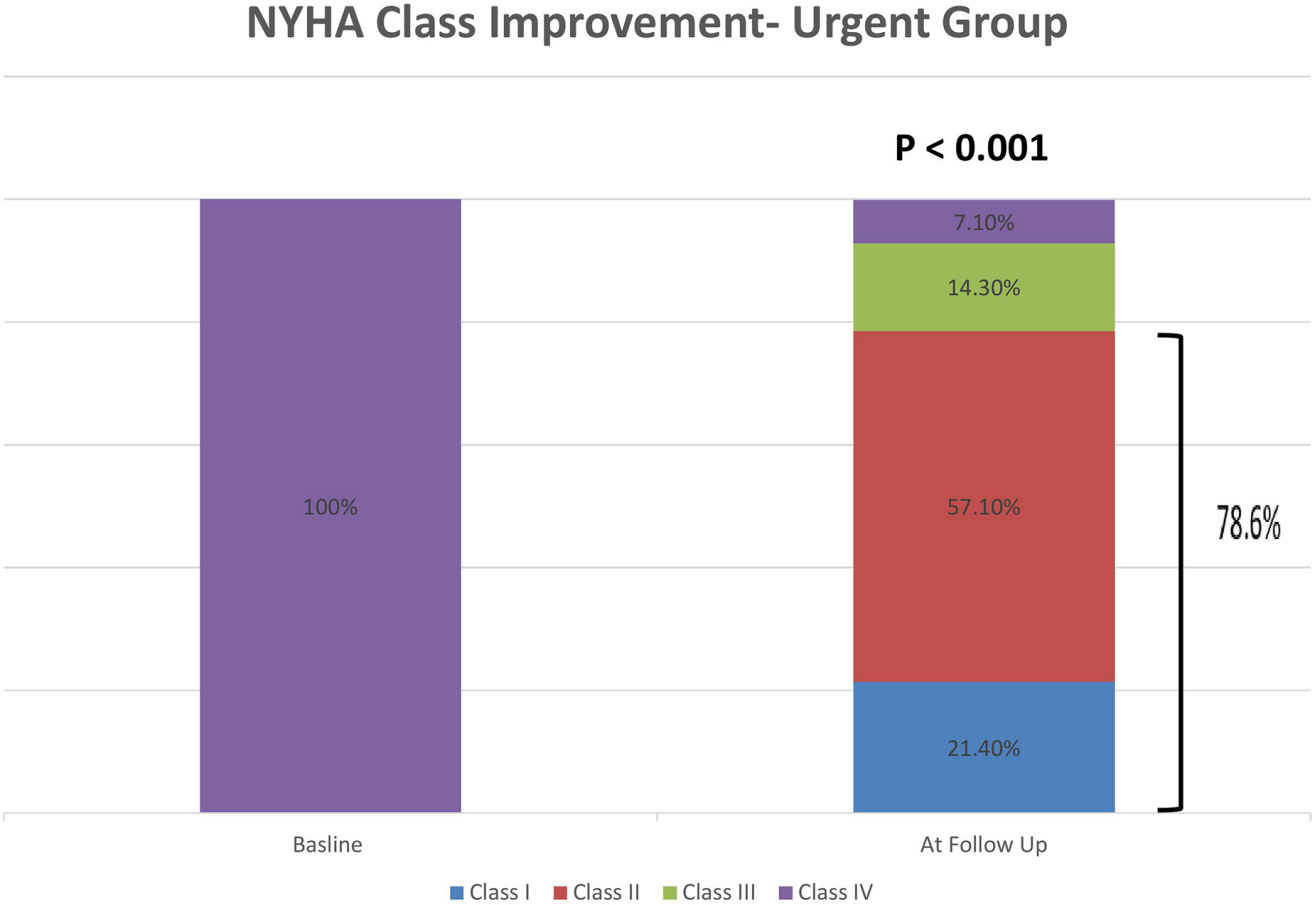
NYHA class before the procedure and after the procedure at a follow up evaluation in the urgent group. NYHA—New York Heart Association.

There was significant clinical improvement of NYHA class in the urgent (Figure 6), with 11 patients (78%) improving to NYHA I or II at the first follow-up visit (*p* < 0.001). Sixteen patients in the urgent group (94.1%) were alive at least six months following the procedure. There was one periprocedural death in the urgent group due to tamponade following transeptal puncture (Table 4). The elective and urgent groups had similar degrees of NYHA class improvement.

**Table 4:** Clinical outcome of patients in all groups.

	All patients	Elective patients	Urgent patients	*P* Value
NYHA Class at Follow Up (%)	41	27	14	0.551
I	8 (19.5%)	5 (18.5%)	4 (21.4%)	
II	26 (63.4%)	18 (66.7%)	8 (57.1%)	
III	6 (14.6%)	4 (14.8%)	4 (14.3%)	
IV	1 (2.4%)	0 (0%)	1 (7.1%)	
Post Procedure Hospitalization Days (median [IQR])	2.00 [3.0]	1.00 [2.0]	4.00 [3.0]	0.005
Overall Mortality (%)	1 (2.1%)	0 (0%)	1 (5.9%)	0.166
6 Month Survival (%)	48 (97.9%)	100%	16 (94.1%)	0.166
Follow—Up Time—Month (mean [SD])	17.16 (±8.95)	17.75 (±9.24)	16.06 (±8.5)	0.534

NYHA, New York Heart Association; SD, standard deviation.

## Discussion

The natural history of MR due to flail leaflet carries a high risk of morbidity and mortality as a result of left ventricular dysfunction, heart failure, and death. Conservative, non-surgical therapy has been associated with worse clinical outcomes than surgery in this patient population (11, 12).

While the mitral valve surgery has long been the standard of care for patients with flail leaflets, TEER is emerging as a safe and effective alternative in high-risk patients. Current guideline recommendations pertain mainly to patients with chronic MR. For patients with acute MR, recommendations are mainly based on specific pathological conditions such as papillary muscle rupture or infective endocarditis, in which prompt surgical therapy is essential for survival (1, 7). However, patients with acute MR often carry high surgical risk, and many cannot undergo surgery. According to Lorusso et al. who evaluated the outcomes of 279 patients who underwent emergency surgery for severe acute MR caused by acute myocardial infarction, degenerative mitral valve disease, or acute endocarditis, the 30-day mortality rate was 22.5% overall, and 14.8% among patients with degenerative valve disease. The high mortality rates in patients with DMR, who often have preserved left ventricle systolic function and no severe comorbidities, emphasizes the substantial risk of surgery in the acute setting and raises the need for a lower risk intervention such as TEER (6).

In this study we demonstrate and characterize the echocardiographic, hemodynamic, and clinical outcomes of 49 elderly patients (average age was 80 years old) with DMR due to flail leaflet who underwent TEER. We further divided the cohort of patients into patients requiring an acute, urgent TEER procedure, and patients with chronic symptomatic MR who underwent elective procedures. We compared outcomes between the elective and urgent groups, and then focused on the urgent group, describing in more detail their clinical and echocardiographic parameters. In this group, we compared parameters before and after the procedure, aiming to show and emphasize the high rate of procedural success and ultimately their positive clinical outcomes. We showed the procedure to be safe and effective, with a high rate of procedural success in this high-risk elderly population.

Our clinical and echocardiography outcomes were similar to other retrospective studies involving patients with chronic DMR who were at a high surgical risk and underwent TEER (13, 14). However, in our study we exclusively described patients with flail leaflet rather than any other degenerative pathology. The flail mechanism usually denotes severe MR and often implies a more complicated procedure (e.g., large coaptation gap, etc.) (5). The use of newer devices such as the fourth generation Mitraclip or Pascal ACE, (Edwards Lifesciences, Irvine, CA) and the ability to perform independent leaflet grasping, allowed treatment for more complex mitral valve anatomies including wide coaptation gaps, large redundant tissue, and large flail gap.

In contrast to chronic MR, in which there is time for a compensating mechanism and thus a more gradual clinical deterioration, acute MR patients typically present with severe respiratory distress, often with pulmonary edema and cardiogenic shock (15).

There are currently less data on TEER in patients with acute MR, although emerging data shows favorable results in patients with functional MR (mostly secondary to ischemic insult) (16–18). There is limited information describing patients with acute primary MR due to ruptured chordae who undergo acute TEER procedures.

The average age and comorbidities of our cohort were similar to previously mentioned DMR patients who underwent TEER. However, their acute presentation and severe current illness put them at a higher risk for surgery and overall mortality. We demonstrate a high rate of MR reduction, with most patients having >2 grade reduction. This reduction in the severity of MR addresses the main concern regarding percutaneous repair vs. surgical repair, where surgical repair has previously been shown to achieve greater MR reduction than TEER (19). Furthermore, we showed a significant clinical benefit by improving the patient's NYHA class. This clinical improvement supports the previous finding that procedural success with significant MR reduction is the major determinant of clinical outcome (20, 21).

The mortality rate of patients undergoing urgent TEER was non-inferior to the mortality rate of the elective patients with chronic MR in our study. These findings further support the safety and clinical benefit of TEER in patients considered high risk for surgery.

### Limitation

This analysis is based on retrospective observational data without a control group and is limited in size. In a larger group of patients results might be different. Because the study cohort included patients undergoing TEER at a high-volume single center with an experienced team, the results of the procedure and the outcomes of the patients cannot be generalized to all other centers performing TEER. Fifty percent of the patients from the urgent group were referred from different hospital. Based on the available data of these patients, the average time from presentation to the procedure was more than 10 days. This might represent a selection bias, since patients referred to the procedure have survived long enough to undergo the procedure and benefit from repairing their MR. On contrary, patients that were too unstable and could not survive long enough to undergo the procedure may not be included in the study cohort.

Several patients that were referred from other hospitals had incomplete data that could not be included in our study analysis. Our findings support the role of TEER in these acutely ill elderly patients who do not have other therapeutic options. One potential disadvantage for TEER is that patients with suboptimal results who require subsequent valve surgery can only get mitral valve replacement, and not repair due to the presence of TEER devices.

Finally, the evaluation of procedural success in patients after TEER device implantation can be challenging, even more so in patients with acute MR where doppler evaluation is more limited. Although the standard evaluation of MR reduction is made by doppler evaluation, our finding emphasizes the limited ability of color doppler alone to fully evaluate the whole hemodynamic effect of the MR reduction, particularly in our cohort. Nearly 40% of our patients had moderate MR after the procedure, but their hemodynamic parameters showed more impressive improvements. The V wave reduction after the procedure was reduced by an average of 20 mmHg, the pulmonic vein flow normalized in most of the patients and ultimately the improvement of NYHA class supported the success of the procedure beyond the reported grade of MR by color flow.

## Conclusion

TEER can be safe and effective in patients with DMR, including patients presenting with acute severe MR. In acutely ill MR patients with high surgical risk, TEER can serve as a good therapeutic alternative, providing a bridge to recovery.

## Data Availability

The raw data supporting the conclusions of this article will be made available by the authors, without undue reservation.
